# Shortening effect on spinal canal and clinical results by conversion of kyphosis into lordosis by Pedicle Subtraction Osteotomy (PSO) at the thoracolumbar junction in spinal deformities

**DOI:** 10.1186/1748-7161-9-S1-O54

**Published:** 2014-12-04

**Authors:** Piet van Loon, Martijn Mantingh, Nico Verdonschot

**Affiliations:** 1 Gelre Ziekenhuis, Apeldoorn, Netherlands; 2 University Medical Centre, Groningen, Netherlands; 3 Radboud University, Nijmegen, Netherlands

## Background context

PSO can restore sagittal balance in kyphosis and scoliosis. Effect on central cord-root system seems unknown.

## Purpose

To measure shortening of canal in PSO as side effect and describe macroscopic and MRI changes in the cord.

## Study design

Radiographic study of the canal-shortening and lordosating effect as feature of a wedge or PSO the TL- spine. A case series with emphasis on radiographic alterations of the canal and clinical results.

## Material

Out of a group of 21 consecutive patients (mean age 42,7 yr (13-69)) who had a fusion inclusive a PSO for kyphotic deformities, clinical and radiological parameters were taken preoperatively and at follow-up ( minimal 12 months). Complications are described.

## Outcome measures

Radiographs were measured pre-and postoperatively. Shortening at the frontal side of the canal in the osteotomised vertebra and between top Th10 to bottom L2 was measured as was the ventral side of the column Th10-L2. Correction of sagittal curve Th10-L2 was measured. Preoperative SF36 and VAS for pain and fatigue were compared with follow-up.

## Methods

21 adults with a kyphotic deformity were operated and followed for 12 to 29 months .

## Results

The height of Th10-L2 at the ventral and at the posterior part of the bodies shows a mean shortening at the ventral side of 2,3mm and 7.6mm at the canal side of the bodies, the osteotomised vertebra taking 98% of the latter value. Correction of sagittal profile of Th10-L2 changed from a mean +18º to a mean -9º. SF-36 improves by a mean of 33,6 (SD17.1) to 49,7(SD 27,4) : p<0.01. VAS for pain improved from 9 (SD 2.6) to 5.5 (SD 2.8)(p<0.01). VAS for fatigue improved from 7.85 (SD 2,2) to 5,45 (SD 2,8) (p<0.01). Two voluntary MRI’s at follow up are adjuvant in explanation of results.

## Discussion and conclusion

In lordosating TLJ by PSO a true shortening effect on the canal is measured. The series shows the appreciation in some parameters by patients and the actual shortening of the spinal canal at the crucial thoracolumbar area by performing a PSO in fixed kyphotic deformities. Indication and mimicking effects surgery vs. TLI bracing will be discussed.

